# Natural brain-information interfaces: Recommending information by relevance inferred from human brain signals

**DOI:** 10.1038/srep38580

**Published:** 2016-12-08

**Authors:** Manuel J. A. Eugster, Tuukka Ruotsalo, Michiel M. Spapé, Oswald Barral, Niklas Ravaja, Giulio Jacucci, Samuel Kaski

**Affiliations:** 1 Aalto University, Department of Computer Science, Helsinki Institute for Information Technology HIIT, P.O. Box 15400, FI-00076 AALTO, Finland; 2 University of Helsinki, Department of Computer Science, Helsinki Institute for Information Technology HIIT, P.O. Box 68, FI-00014 University of Helsinki, Finland; 3 University of Helsinki, Department of Social Research, Helsinki Institute for Information Technology HIIT, P.O. Box 54, FI-00014 University of Helsinki, Finland

## Abstract

Finding relevant information from large document collections such as the World Wide Web is a common task in our daily lives. Estimation of a user’s interest or search intention is necessary to recommend and retrieve relevant information from these collections. We introduce a brain-information interface used for recommending information by relevance inferred directly from brain signals. In experiments, participants were asked to read Wikipedia documents about a selection of topics while their EEG was recorded. Based on the prediction of word relevance, the individual’s search intent was modeled and successfully used for retrieving new relevant documents from the whole English Wikipedia corpus. The results show that the users’ interests toward digital content can be modeled from the brain signals evoked by reading. The introduced brain-relevance paradigm enables the recommendation of information without any explicit user interaction and may be applied across diverse information-intensive applications.

Documents on the World Wide Web, and seemingly countless other information sources available in a variety of online services, have become a central resource in our day-to-day decisions. As our capabilities are limited in finding relevant information from large collections, computational recommender systems have been introduced to alleviate information overload^1^. To predict our future needs and intentions, recommender systems rely on the history of observations about our interests^2^. Unfortunately, people are reluctant to provide explicit feedback to recommender systems^3^. As a consequence, acquiring information about user intents has become a major bottleneck to recommendation performance, and sources of information about the individuals’ interests have been limited to the implicit monitoring of online behavior, such as which documents they read, which videos they watch, or for which items they shop^3^. An intriguing alternative is to monitor the brain activity of an individual to infer information about relevance.

To utilize brain signals, we introduce a brain-relevance paradigm for information filtering. The paradigm is based on the hypothesis that relevance feedback on individual words, estimated from brain activity during a reading task, can be utilized to automatically recommend a set of documents relevant to the user’s topic of interest (see Fig. 1 for an illustration). Following the brain-relevance paradigm, we introduce the first end-to-end methodology for performing fully automatic information filtering by using only brain activity associated with the relevance of individual words. The methodology is based on predicting and modeling the user’s informational intents using brain signals and the associated text corpus statistics and then recommending new and unseen information using the estimated intent model. The methodology involves the human in the retrieval process as a source of relevance feedback^4^ in order to improve the relevance estimated for unseen information. The general idea of relevance feedback is that the human provides feedback on the relevance of the initially presented information and that the retrieval system provides better information based on this feedback. In addition, the predicted relevance is used to infer the informational intentions (i.e., the intent model^5^) of the individual and to retrieve new information that matches these intentions. Instead of directly using the words inferred to be relevant as a search query, we use these words to infer relevance over all words in the system. This distribution of relevance over all words then defines the individual’s informational intentions. A system utilizing the brain-relevance paradigm and using the presented end-to-end methodology can mitigate the requirement of explicit human-computer interaction to convey relevance feedback on individual words directly from brain activity during a reading task.

Unlike the standard active brain-computer interface (BCI) in which the user memorizes, often a motoric pre-defined pattern (e.g., imagine moving your left arm) that the computing system is trained to detect, we utilize passive BCI methodology^6^. Passive BCI methodology relies on reactive states of the user’s cognition automatically induced while interacting in the surrounding system (e.g., an individual naturally reacting to a certain word that is relevant or meaningful to him or her). In our case, the reactive states are naturally evoked by reading relevant words and the user is not required to perform any additional, explicit tasks (e.g., mental counting of relevant words or imaginary motor activity) that have been previously shown to enhance the signal-to-noise ratio^7^. While passive BCI has recently been used in specific applications, such as the detection of emergency braking intention while driving a car^8^ and workload detection^9^, we utilize it, for the first time, for predicting the informational intentions of an individual examining digital textual content.

The data from experiments, in which electroencephalography (EEG) was recorded from 15 participants (of the 17 recorded participants, two were discarded due to technical issues) while they were reading texts, shows that the recommendation of new relevant information can be significantly improved using brain signals when compared to a random baseline. The result suggests that relevance can be predicted from brain signals that are naturally evoked when users read, and they can be utilized in recommending new information from the Web as a part of our everyday information-seeking activities.

## Brain-relevance paradigm for information filtering

We propose a new paradigm for information filtering based on brain activity associated with relevance. The *brain-relevance paradigm* is based on the following four hypotheses evaluated empirically in this paper:

**H1:** Brain activity associated with relevant words is different from brain activity associated with irrelevant words.

**H2:** Words can be inferred to be relevant or irrelevant based on the associated brain activity.

**H3:** Words inferred to be relevant are more informative for document retrieval than words inferred to be irrelevant.

**H4:** Relevant documents can be recommended based on the inferred relevant and informative words.

The following two sections provide motivation based on both cognitive neuroscience and information science, followed by existing foundations of the brain-relevance paradigm.

### Cognitive neuroscience motivation

Event-related potentials (ERPs) are obtained by synchronizing electrical potentials from EEG to the onset (“time-locked”) of sensory or motoric events^10^. The last 50 years of psychophysiology have demonstrated beyond a reasonable doubt that ERPs have a neural origin, that cognitive processes can reliably elicit them, and that the measurement of their timing, scalp distribution (“topography”), and amplitude can provide invaluable information about brain function of healthy^11^ and pathological^12^ cases.

Mentally controlling interfaces through measured ERPs has, to date, principally relied on the P300. The P300 is a distinct, positive potential that occurs at least 300 ms after stimulus onset and is traditionally obtained via so-called oddball paradigms. Sutton *et al*.^13^ presented a fast series of simple stimuli with infrequently occurring deviants (e.g., 1 in 6 tones having a high pitch) and discovered that these rare “oddballs” would on average trigger a positivity compared to the standard stimuli. Experiments later showed that the degree to which the stimulus provided new information^14^ and was task-relevant^15^ amplified the P300, whereas repetitive, unattended^16^, or easily processed^17^ stimuli could remove the P300 entirely.

For the language domain, the onset of words normally evokes a negativity at ca. 400 ms, which has been attributed to semantic processing^18^. This N400 was first observed as a type of “semantic oddball” since the closing word in a sentence, such as “I like my coffee with milk and torpedoes,” is semantically improbable but would amplify the N400 rather than cause a P300. However, if a rare syntactic violation occurs in a sentence (“I likes my coffee [..]”), the deviant word once again evokes a positivity, but now at 600 ms^19^. As this P600 shows similarities to the P300 in polarity and topography, it started the ongoing debate as to whether it is a language-specific “syntactic positive shift” or a delayed P300^20^,^21^,^22^. Finally, research on memory has identified a late positive component (LPC) at a latency similar to the P600. The LPC has been related to semantic priming and is particularly strong in tasks where an explicit judgement on whether a word is old or new is to be made^23^. Consequently, it is often associated with mnemonic operations such as recollection^24^. In the present context, relevant words could cue recollection of the user’s intent, thereby amplifying the LPC.

Although the P300/P600 and N400 are often described as contrasting effects, this is not necessarily the case in predicting term relevance. That is, if an odd, task-relevant stimulus yields a P300 or P600 and a semantically irrelevant stimulus an N400, it follows that the total amount of positivity between an estimated 300 and 700 ms may indicate the summed total semantic task relevance. This was indeed found by Kotchoubey and Lang^25^, who showed that semantically relevant oddballs (animal names) that were randomly intermixed among words from four other categories evoked a P300-like response for semantic relevance (but at ca. 600 ms). Likewise, our previous work on inferring term relevance from event-related potentials^26^, showed that a search category elicited either P300s/P600s in response to relevant words or N400s evoked by semantically irrelevant terms.

### Information science motivation

Relevance estimation aims to quantify how well the retrieved information meets the user’s information needs. Computational methods are used in estimating statistical relevance measures based on word occurrences in a document collection. These measures are used in many information retrieval applications, such as Web search engines, recommender systems, and digital libraries. One of the most well-known statistical measures for this purpose is *tf-idf*, which stands for term frequency-inverse document frequency. The *tf-idf* weight is a weight often used in information retrieval to statistically measure how important a word is to a document in a collection or corpus. We use a logarithmically scaled *tf-idf* formally defined in SI Equation 3 to SI Equation 5. The importance of a word increases proportionally to the number of times a word appears in the document but is offset by the frequency of the word in the corpus; low- and medium-frequency words have a higher discriminating power at the level of the document collection, particularly when they have high frequency in an individual document^27^. For example, the word “nucleus” has a low frequency at the collection level (i.e., across all documents in the system) but a higher frequency in a document about atoms (i.e., the “Atoms” document) and is therefore considered to discriminate this document better than, for example, the word “the,” which has a high frequency at both the collection and document levels.

Consequently, relevance feedback for different words has a different effect in predicting an individual’s search intention. For example, detecting that an individual finds the word “the” relevant is less valuable than detecting the relevance of the word “nucleus” because the latter has a higher discriminative power and can be more effectively used to predict the individual’s intention of finding documents, such as the “Atoms” document.

In summary, the brain-relevance paradigm requires the relevance of an individual word to be inferred from the individual’s brain signals. Word informativeness is determined by the search system using the *tf-idf* statistic. Words that are both relevant and informative are words that discriminate relevant documents from irrelevant documents and are needed to predict the individual’s search intention and, consequently, to recommend meaningful documents. In addition to the brain activity findings related to the semantic oddball (introduced in the cognitive neuroscience motivation), recent findings in quantifying brain activity associated with language also suggest a connection between the word class and frequency of the word as well as the corresponding brain activity. It has been shown that brain activity is different for different word classes in language^28^ and that high-frequency words elicit different activity than low-frequency words^29^.

## Methodology

During the experiment, we recorded the EEG signals of 15 participants while each participant performed a set of eight reading tasks. Experimental details are provided in Supplementary Information (SI Neural-Activity Recording Experiment).

### Reading task

The text content read by the participant consisted of two documents at a time. Each document was chosen from a list of 30 candidate documents, and each document was selected from a different topical area. For example, the documents “Atom”, “Money,” and “Michael Jackson” were part of the list; Supplementary Table 1 provides a detailed list of the documents. One document represented the *relevant topic*, the other one an *irrelevant topic*. In the reading task, a document was defined as the first six sentences of a Wikipedia article (i.e., the first sentences of the general introductory paragraph to the topic described in the article). The mean number of words per sentence was 20.05 and the standard deviation was 6.94. The mean number of words per document was 120.31 and the standard deviation was 17.36. The user chose the relevant topic herself at the beginning of the reading task. The user read the sentences from each document–first the opening sentence from both documents, then the second sentence from both documents, and so on (Supplementary Figure 1 provides a step-by-step of the experiment and Supplementary Figure 2 provides an illustration of its technical implementation). The obtained brain signals were used to predict term-relevance feedback, which was then used to retrieve further documents relevant to the user-chosen topic of interest, from among the four million documents available in the database.

**Figure 2:**
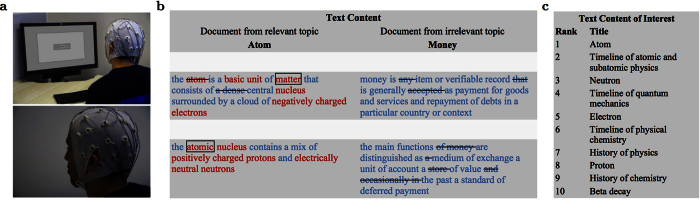
Extract from one experiment to illustrate a reading task with subsequent document retrieval: (**a**) Our data acquisition setup with one participant wearing an EEG cap with embedded electrodes. (**b**) Sample text with the first two sentences from the Wikipedia document “Atom” (relevant document) and the document “Money” (irrelevant document). The color of the words shows the explicit relevance judgments by the user (red: relevant; blue: irrelevant). The crossed-out words were lost because of too much noise in the EEG (e.g., because of eye blinks). The framed words “matter” and “atomic” were the top words predicted to be relevant by the EEG-based classifier. Colors and markings were not shown to the user. (**c**) The top 10 retrieved documents, based on the predicted relevant words, are highly related to the relevant topic “Atom”.

In order for the task to be representative of natural reading, no simplifications were done on the text content. In particular, this implies that the sentences have different numbers of words and that the word length ranges from very short to very long. Figure 2 illustrates one reading task consisting of the relevant document “Atom” and the irrelevant document “Money” with subsequent document retrieval.

### Data analysis

To associate brain activity with relevance, we computed the neural correlates of relevant and irrelevant words for all participants. A participant-specific single-trial prediction model^30^ was computed for each participant, and the performance was evaluated on a left-out reading task (leave-one-task-out, a cross-validation scheme). This procedure matches the example of the task illustrated in Fig. 2, consisting of the following steps: (1) users perform a new reading task; (2) relevance predictions are made for each word based on a model that was trained on observations collected during previous reading tasks; and (3) documents are retrieved using the relevance predictions for the present reading task. We present results for the (H1) neural correlates of relevance, (H2) term-relevance prediction, (H3) relation between relevance prediction and word importance, and (H4) document recommendation. Results are presented for both individual users and as grand averages. Technical details are in Supplementary Information (SI Data Analysis Details).

### Evaluation

To quantify the significance and the effect sizes of the *brain feedback*-based prediction performances, we compared them against performances from prediction models learned from *randomized feedback*. By comparing against this baseline, we are able to operate with natural and, hence, non-balanced texts. Standard permutation tests^31^ were applied for significance testing.

We used the *Area under the ROC curve* (AUC) to quantify the performance of the classifiers. AUC is a widely used and sensible measure, even under the class imbalances of our scenario, and it is a comprehensive measure for comparison against the prediction models based on randomized feedback. From the perspective of document recommendations, it is more important to predict relevant words than to predict irrelevant words. To quantify this, we measured the *precision* (Supplementary Information, Equation 1). To demonstrate the influence of a positive predicted word on the document retrieval problem, we additionally measured the *tf-idf-weighted precision* (Supplementary Information, Equation 2). From the user perspective, the quality of the recommended documents is important. To quantify this, we used *cumulative information gain*, which measures the sum of the graded relevance values of the returned documents (Supplementary Information, Equation 7). AUC and precision are based on participant-specific relevance judgments, and cumulative information gain is based on external topic-level expert judgments. Details on the concrete definition of the evaluation measurements and the assessment process are available in the Supplementary Information.

## Results

### Neural correlates of relevance

**Figure 3:**
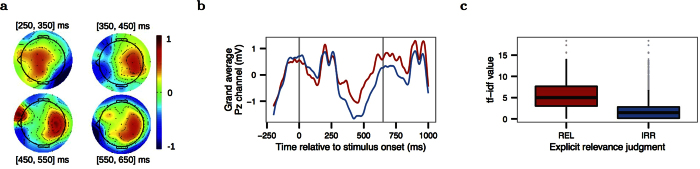
Grand average results over all participants and reading tasks based on explicit relevance judgments: (**a**) Grand average-based topographic scalp plots of relevant minus irrelevant ERPs from [250, 350] ms, [350, 450] ms, [450, 550] ms, and [550, 650] ms after word onset. (**b**) Grand average event-related potential at the Pz channel of relevant (red curve) and irrelevant (blue curve) terms. The gray vertical lines show the word onset events. (**c**) Term frequency-inverse document frequency values (*tf-idf*) of relevant (red box plot) and irrelevant (blue box plot) words. The median of relevant words is 5.00, and that of irrelevant words is 1.46. The difference is significant (Wilcoxon test, *V* = 49680192, *p* < 0.0001).

Grand-average-based ERP results show that brain activity associated with relevant words is different from brain activity associated with irrelevant words (H1) over all participants and reading tasks. The topographic scalp plots in Fig. 3a show the spatial interpolation of relevant ERPs minus irrelevant ERPs over all electrodes from 300 ms to 600 ms after a word was shown on screen. The topography of the difference showed an initial fronto-central positivity at 300 ms, relative to the onset of the word on the screen, followed by a centro-parietal positivity from 400 to 600 ms. The maximal effect of relevance can be clearly observed in Fig. 3b, with −0.24 *μ*V for relevant words and −1.06 *μ*V for irrelevant words at 367 ms over Pz. Following the negativity, a late positivity can be observed for both types of words, which reaches a local maximum at a latency of around 600 ms, thus implicating a possible P600 or LPC.

For descriptive purposes, we tested the difference between the relevant and irrelevant words of well-known P300, N400, and P600 ERP components and their latencies given in the existing literature. There was no significant difference in the early P3 interval ([250, 350] ms, paired *t*-test, *T*(14) = 1.75, *p* = 0.10), which suggests that the system does not rely on the mere visual resemblance between relevant words and the intent category. However, irrelevant words elicited a negativity compared to relevant words in the N400 window ([350, 500] ms, *T*(14) = 2.27, *p* = 0.04). Moreover, relevant words were found to significantly elicit a positivity compared to irrelevant words in the P600 interval ([500, 850] ms, paired *t*-test interval, *T*(14) = 4.99, *p* = 0.0002). For the purpose of the subsequent term-relevance prediction, this result verified our approach of computing the temporal features for the ERP classification within the range of 200 ms to 950 ms (this range was determined based on the pilot experiments). Supplementary Figure 1 shows the remaining scalp plots for other time intervals, and Supplementary Figure 2 shows the grand-average-based ERP curves for all channels.

### Term-relevance prediction

**Figure 4:**
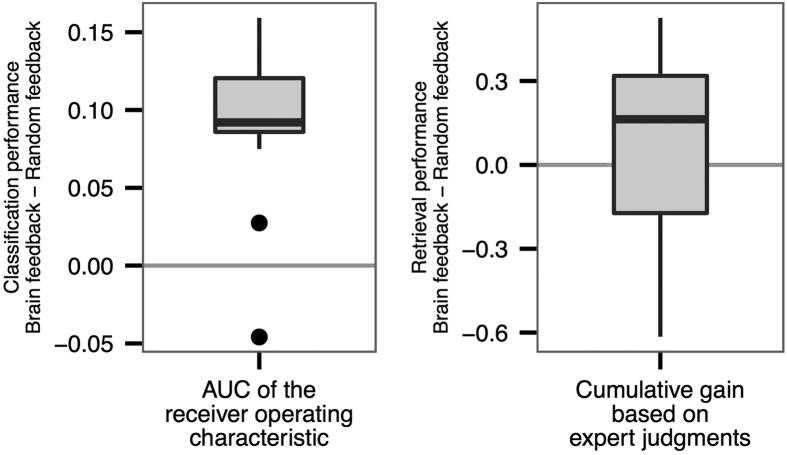
Overall prediction and retrieval performances: (**a**) Overall classification performance on new data, measured by the difference of AUC between a classifier learned using explicit relevance feedback and that learned using randomized feedback. The difference is significantly greater than zero (*p* < 0.0005). The figure shows that the prediction models are able to find structures significantly discriminating between relevant and irrelevant brain signal patterns. (**b**) Overall retrieval performance characterized as the difference in cumulative gain (based on expert judgments) between documents retrieved based on brain-based feedback and randomized feedback (normalized with the maximum information gain that would be possible to achieve when retrieving the best top 30 documents). Brain feedback is significantly better than randomized feedback (*p* < 0.003).

Across participants and reading tasks, the classification of brain signals by models learned from earlier explicit feedback shows significantly better results than with models learned from randomized feedback (Fig. 4a; *p* < 0.0005, Wilcoxon test, *V* = 118). This implies that the prediction models are able to extract and utilize structured signals significantly and that words can be inferred to be relevant or irrelevant based on the associated brain activity (H2).

**Figure 5:**
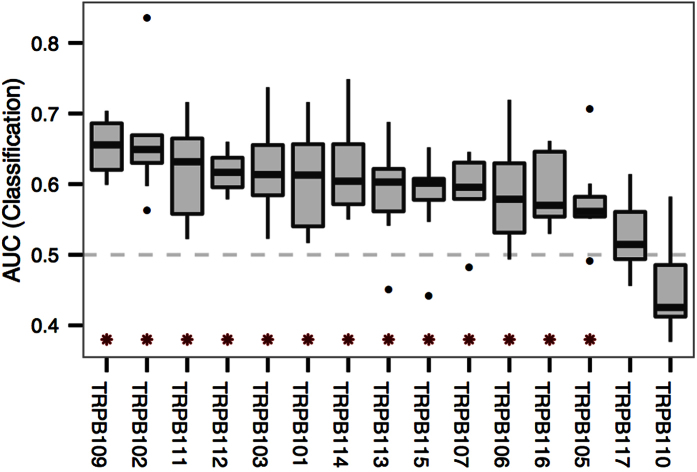
Comprehensive term-relevance prediction performance on participant level: classification performance for all participants (TRPB#), measured as AUC for participant-specific models on left-out reading tasks. The horizontal dashed line indicates the performance of a model learned using randomized feedback. Asterisks indicate models with significantly better AUC (*p* < 0.05; exact *p*-values are listed in Supplementary Table 3).

Figure 5 shows the classification performance in terms of AUC for each participant. For 13 out of the 15 participants, the term-relevance prediction models perform significantly better than a prediction model learned based on randomized feedback (hence having AUC > 0.5; *p* < 0.05, within-participant permutation tests with 1000 iterations). For two participants, the predictions were essentially random (AUC not significantly better than 0.5) and they were excluded from the rest of the analyses. It is well known that BCI control does not work for a non-negligible portion of participants (ca. 15–30%^32^), and the reported results should be interpreted as being valid for the population of users, which can be rapidly screened by using the system on pre-defined tasks.

### Relevance for document retrieval

**Figure 6:**
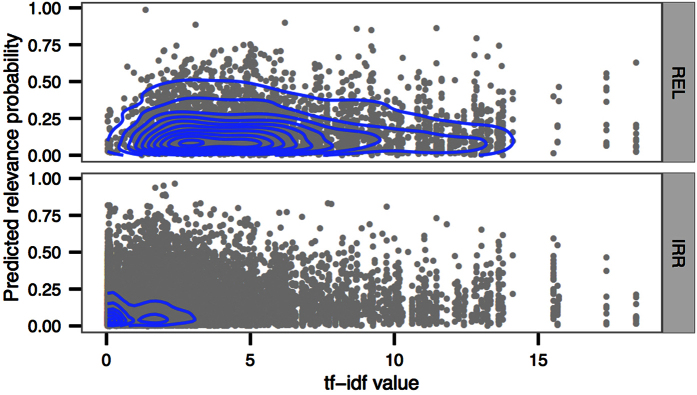
Relevance prediction versus *tf-idf* value: Two-dimensional kernel density estimate (the blue contours) for relevant (top) and irrelevant (bottom) words with an axis-aligned bivariate normal kernel. The mass of relevant words (REL) is much more toward the top right corner (high probability to be relevant and high *tf-idf* value) than the mass of irrelevant words (IRR). The gray points in the background are the observed words.

For our final goal, which is the retrieval and recommendation of documents, it is important to be able to detect words that are both relevant and informative (measured by the *tf-idf*) in discriminating between relevant and irrelevant documents in the full collection. Figure 6 visualizes the relationship between the predicted relevance probability of words and their *tf-idf* values. Relevant words (according to the user’s own judgement afterwards) are predicted as being more relevant than irrelevant words, but their *tf-idf* values are also greater (H3). The figure further indicates that the *tf-idf* dimension explains more of the difference than the predicted relevance.

**Figure 7:**
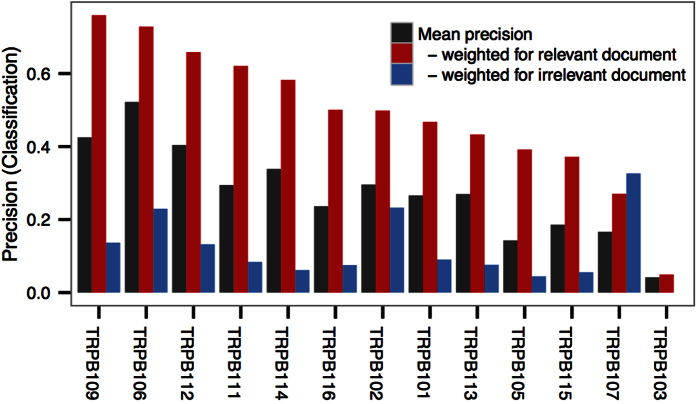
Relevance prediction weighted for retrieval on participant level (for the 13 participants with term-relevance prediction models performing significantly better than random feedback prediction models): Mean precision per participant (black bars). For the document retrieval problem, the influence of a positive predicted word is dependent on its document-specific *tf-idf* value. Therefore, a false positive can have a smaller effect than a true positive. The red and blue bars illustrate this effect. The red bars show the precision weighted with the *tf-idf* value of the relevant document. The blue bars show the precision weighted with the *tf-idf* value of the irrelevant topic.

In terms of an information retrieval application, the precision of the prediction models is the most important measure. For document retrieval, the influences of positive predicted words on the search results are not equal but dependent on the word-specific *tf-idf* values within each individual document. For example, a true positive predicted word can still have very low impact on the search result if its *tf-idf* value is low in the relevant documents. Similarly, a false positive predicted word can only have a low impact on the search result if its *tf-idf* value is low. Figure 7 visualizes the mean precision of the prediction models from the perspective of the retrieval problem. It shows the mean precision for each of the 13 participants over all of the reading tasks (based on binarizing the predicted probabilities with the threshold 0.05). In addition, it shows what is actually crucial: The precision weighted with the *tf-idf* values from the relevant document is, in all cases except for one, much higher than the precision weighted with the *tf-idf* values from the irrelevant document.

In conclusion, the results in Fig. 7 explain why the prediction models are useful for document retrieval and recommendation, even though the unweighted precision of the prediction models is limited. In detail, our prediction models tend to predict true positive words with higher *tf-idf* values and false positive words with lower *tf-idf* values. This means that our prediction models tend to predict words that the user would judge to be relevant and which are also discriminative in terms of the user’s search intent.

### Document recommendation

The final step is to use the relevant words–predicted from brain signals–for document retrieval and recommendation and to evaluate the cumulative gain. Figure 4b shows that across the participants and reading tasks, document retrieval performance based on brain feedback is significantly better than randomized feedback (top 30 documents, *p* < 0.003, Wilcoxon test, *V* = 3153). Therefore, relevant documents can be recommended based on the inferred relevant and informative words (H4).

**Figure 8:**
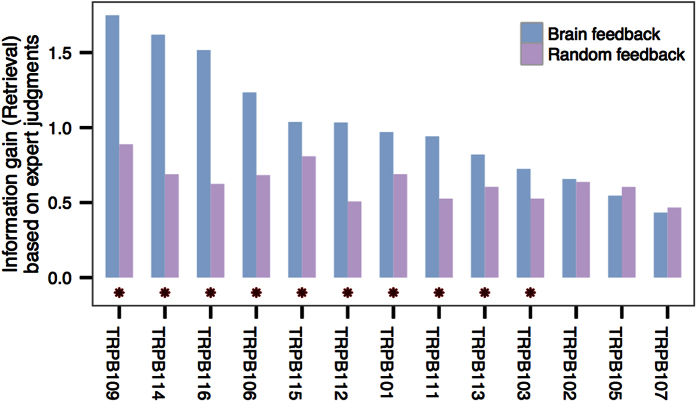
Retrieval performance of each participant (for the 13 participants with term-relevance prediction models performing significantly better than random feedback prediction models): Average cumulative information gain (on a scale of 0–3) based on the top 30 retrieved documents for the participant. Asterisks indicate a significantly better pooled information gain based on brain feedback than random feedback retrieval based on 1000 iterations (*p* < 0.05; exact *p*-values are listed in Supplementary Table 4).

Figure 8 shows the document retrieval performance for each participant in terms of mean information gain. Based on the expert scoring, the scale for the mean information gain is from 0 (irrelevant) to 3 (highly relevant). For each participant, the visualization shows the mean information gain over all reading tasks based on brain feedback (blue bars) and randomized feedback (purple bars). For 10 participants, the brain feedback results in significantly greater information gain (*p* < 0.05; two-sided Wilcoxon test). Supplementary Figure 3 also shows the visualizations for the top 10 and top 20 retrieved documents. In both cases, the same significant results hold except for one participant (TRPB113).

## Discussion

By combining insights on information science and cognitive neuroscience, we proposed the brain-relevance paradigm to construct maximally natural interfaces for information filtering: The user simply reads, brain activity is monitored, and new information is recommended. To our knowledge, this is the first end-to-end demonstration that the recommendation of new information is possible by only monitoring the brain activity while the user is reading.

The brain-relevance paradigm for information filtering is based on four hypotheses empirically demonstrated in this paper. We showed that (H1) there is a difference in brain activity associated with relevant versus irrelevant words; (H2) there is a difference in the importance of words depending on their relevance to the user’s search intent; (H3) it is possible to detect relevant and informative words based on brain activity given the task/context; and (H4) it is possible to recommend relevant documents based on the detected relevant and informative words.

From a cognitive neuroscience point of view, it is known that specific ERPs can be particularly associated with relevance. In cognitive science, early P300s have been related with task relevance. In psycholinguistics, N400s are commonly associated with semantic processes^18^, as semantically incongruent words amplify the component whereas semantic relevance reduces it. Late positivity has been related to semantic task-relevant stimuli^25^, particularly if characterizing it as a delayed P3 response due to the assessing of relevance of language, or an LPC, due to mnemonic operations and semantic judgements. In line with these findings, our grand averages indicate that the ERP at a latency of 500–850 ms is most likely the best predictor of perceiving words that are semantically related to a user’s search intent. The present data do not allow for a dissociation among the P300, N400 or P600 as the most likely neural candidate for evoking the observed effect. Indeed, the method is based on the assumption that task relevance and semantic relevance both contribute positively to the inference of relevance when aiming to ultimately predict a user’s search intent without requiring an additional task by the user.

While our results use real data and are also valid beyond the particular experimental setup, our methodology is limited to experimental setups in which it is possible to control strong noise, such as noise due to physical movements, which are known to cause confounding artifacts in the EEG signal. Another limitation is that the comparison setup in our studies considers only two topics at a time: one being relevant and another being irrelevant. While this is a solid experimental design and can rule out many confounding factors, it may not be valid in more realistic scenarios in which users choose among a variety of topics during their information-seeking activities. Furthermore, the presented term-relevance prediction is based on a traditional set of event-related potentials to demonstrate the methodology’s feasibility. However, it is possible that more advanced feature extraction could improve the solution further, for example, by computing phase synchronization statistics in the delta and theta frequencies, which have recently been shown to be sensitive to the detection of relevant lexical information^33^.

Despite these limitations, our work is the first to address an end-to-end methodology for performing fully automatic information filtering by using only the associated brain activity. Our experiments demonstrate that our method works without any requirements of a background task or artificially evoked event-related potentials; the users are simply reading text, and new information is recommended. Our findings are a step toward systems that analyze relevance directly from individuals’ brain signals naturally elicited as part of our everyday information-seeking activities.

## Materials and Methods

The Supplementary Information provides extensive details on all technical aspects. SI Database describes the selection process and the criteria for the pool of candidate documents. SI Neural-Activity Recording Experiment provides the experimental details (i.e., the participant recruiting, the procedure and design of the EEG recording experiment, the apparatus and stimuli definition, and details on the pilot experiments). SI Data Analysis Details describe the general prediction evaluation setup, EEG pre-processing, and the EEG feature engineering. SI Term-Relevance Prediction provides a description of the Linear Discriminant Analysis (LDA) method used for the prediction models and the specifics on the evaluation measures for prediction. SI Intent Modeling-Based Recommendation provides details on the intent estimation model based on the LinRel algorithm and specifics on the evaluation measures for document retrieval.

The study was fully designed and performed in accordance with the relevant guidelines and regulations, particularly those set out in the Declaration of Helsinki pertaining to the ethical treatment of human subjects. Participants were fully briefed as to the nature and purpose of the study prior to the experiment, signed informed consents, and were instructed on their rights as participants, including the right to withdraw from the experiment at any time without fear of negative consequences. All experimental protocols were approved by the University of Helsinki Ethical Review Board in the Humanities and Behavioural Sciences.

## Additional Information

**How to cite this article**: Eugster, M. J. A. *et al*. Natural brain-information interfaces: Recommending information by relevance inferred from human brain signals. *Sci. Rep.* **6**, 38580; doi: 10.1038/srep38580 (2016).

**Publisher’s note:** Springer Nature remains neutral with regard to jurisdictional claims in published maps and institutional affiliations.

## Supplementary Material

Supplementary Information

## Figures and Tables

**Figure 1:**
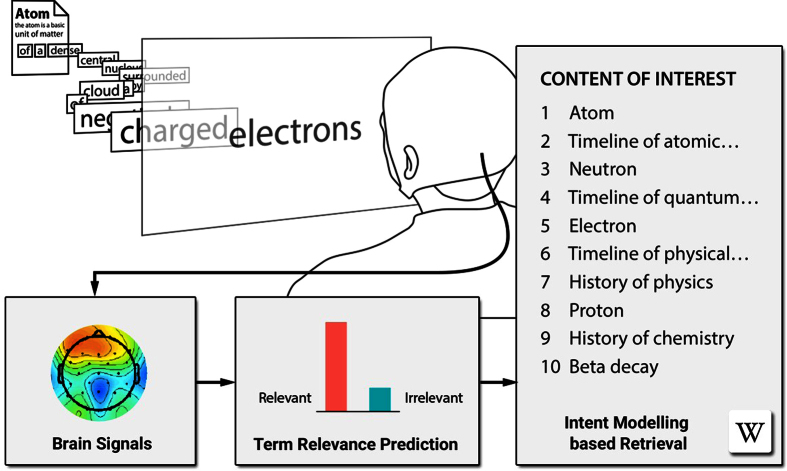
The user reads text from the English Wikipedia while the event-related potentials (ERPs) are recorded using electroencephalography (EEG). A classifier is trained to distinguish the relevant from the irrelevant words by using the ERPs associated with each word in the text. An intent model uses the relevance estimates as input and then infers the user’s search intent. The intent model is used to retrieve new information from the English Wikipedia. (We thank Khalil Klouche for designing the figure).
