# Supplementary material for: Natural brain-information interfaces: Recommending information by relevance inferred from human brain signals
Source: Sci Rep. 2016 Dec 8;6:38580. doi: 10.1038/srep38580 (PMC5143956; doi:10.1038/srep38580)
Supplement: Supplementary Information [file srep38580-s1.pdf]

Supplementary Information SI

Natural brain-information interfaces: Recommending information by relevance inferred from human brain signals

Manuel J. A. Eugster[1,+], Tuukka Ruotsalo[1,+], Michiel M. Spapé[1,+], Oswald Barral[2], Niklas Ravaja[1,3], Giulio Jacucci[1,2], and Samuel Kaski[1,2,*]

[1]Aalto University, Department of Computer Science, Helsinki Institute for Information Technology HIIT, P.O. Box 15400, FI-00076 AALTO, Finland.
[2]University of Helsinki, Department of Computer Science, Helsinki Institute for Information Technology HIIT, P.O. Box 68, FI-00014 UNIVERSITY OF HELSINKI, Finland.
[3]University of Helsinki, Department of Social Research, Helsinki Institute for Information Technology HIIT, P.O. Box 54, FI-00014 UNIVERSITY OF HELSINKI, Finland.

SI Database

The database used in the experiment was the English Wikipedia provided by Wikimedia (database dump of 2014/07/07[1]). For the experiment, our search engine indexed all articles except special pages such as disambiguation pages. The references, notes, and external links were removed from the text of the articles. The final database contained over 4 million articles.

The *pool of candidate documents* read by the participants during the experiment consisted of 30 documents. The criteria for choosing a document were that (1) the document should describe a topic of general interest and that (2) the first six sentences of the introduction of the document provide a sufficient description of the topic. The final pool of documents fulfilling these criteria are listed in Supplementary Table1.

SI Relevance Judgments of Words and Documents

In order to measure the relevance prediction performance, "ground truth" in the form of relevance judgements for individual words is needed, for a specific reading task, on both relevant and irrelevant document. The binary relevance judgment "relevant" or "irrelevant" of a word was provided by each participant during the experiment (see SI Neural Activity Recording Experiment). This allowed us to capture the subjective nature of perceived relevance. In addition, for each document, the word class of each word was defined (nouns, verbs, adjectives, etc.) and three experts judged each word as being "relevant" or "irrelevant" for the given document.

In order to measure the document retrieval performance, the "ground truth" relevance judgments of retrieved documents given a relevant topic of the reading task is needed. For each of the 30 documents in the pool, three experts judged all documents that were retrieved in any experiments (brain feedback-based and random feedback-based), resulting in a pool of 13971 retrieved documents. The experts assessed all the documents according to the following criterion: "Would you be satisfied in having this document in the search result list of documents after examining document x? If yes, how satisfied from 1 to 3, if no 0." The mean Cohen's Kappa[2] indicated substantial agreement between the experts, Kappa $= 0.72$.

SI Neural Activity Recording Experiment

We recorded the electroencephalography (EEG) signals of 17 participants while each participant performed a set of eight reading tasks. The following sections provide the experimental details.

Participants

Participants were volunteers recruited from the universities of the Helsinki metropolitan area in Finland. They were selected only if they were right-handed, had no self-reported neuropathological history, and were deemed to have sufficient fluency in English. Handedness was assessed using the Edinburgh Handedness Inventory[3,4] and English fluency using the Cambridge English "Test your English – Adult Learners" online test[5]. Seventeen participants were recruited to participate in the experiment. The data of two participants were discarded due to technical issues. Of the fifteen remaining, 8 were female and 7 male. Their

English fluency was assessed as high (Mean = 23.53, SD = 1.23; maximum value is 25), and their handedness as right-handed (Mean = 87.35, SD = 12.13; the index range between -100 which is fully left-handed to $+100$ which is fully right-handed). They were fully briefed as to the nature and purpose of the study prior to the experiment. Furthermore, and in accordance with the Declaration of Helsinki, they signed informed consents and were instructed on their rights as participants, including the right to withdraw from the experiment at any time without fear of negative consequences. They received two movie tickets as compensation for their participation.

Procedure and Design

Following the initial briefing, participants were explained the task in more detail, while the EEG equipment was set up. They then received a short training task with two sample topics. When participants indicate their complete understanding of the task, the experiment commenced. One experimental block was called a *reading task*. During one reading task two documents were read. The two documents were randomly drawn (without replacement) from the pool of 30 document candidates. A document was defined as the first six sentences of the corresponding Wikipedia article. Participants completed eight reading task.

Figure 1 shows the step-by-step explanation of a reading task (also called a block in the visualization) each participant received during the initial briefing. At the beginning of one reading task participants were asked to freely choose which one of the two documents should be the relevant topic and which one the irrelevant topic. Every reading task comprised six trials, each consisting of one sentence form the relevant and one sentence from the irrelevant document. Each trial consisted of the sequential presentation of words (the word stream), which the participants should "just read" and during which their brain signals were monitored. After that, two validity sub-tasks had to be fulfilled to ensure the active participation of the participants. Finally, in the explicit word relevance judgment task, the participants rated the "just read" words as "relevant" or "irrelevant". The explicit judgment task provided the labels for the data analysis. This was needed as we were interested in the subjective relevance of each participant.

Figure 2 shows the concrete implementation of a reading task as a cognitive neuroscience experiment. Every trial started with a warning signal (the words "Starting trial"), followed by the presentation of the mask. An initial sentence separator (a randomized sequence of 4 to 9 numbers or other non-alphabetic characters like `%&$`) was shown before the word stream was shown. The word stream consisted of the sequential presentation of each word in the first sentence, followed by a sentence separator, the words in the second sentence, and concluded by a final sentence separator. Every word and sentence separator was presented for exact 699 ms (SD = 0.3 ms). Punctuation marks were not shown. Masking effects were countered to some extent by the frame resizing, which keeps the level of foveal stimulation constant. In our previous experiment on term-relevance prediction[6] and during the pilot experiments for this experiment, we learned that people had more difficulty reading with than without short masks between the bursts, so as a consequence we removed them. It is possible these masking effects may be much more significant with strong "flashing", as would be the case with very short stimulus durations. Here, the words appearing at a slow rate of ca 700 ms per words. This reading pace was determined in our previous experiment on term-relevance prediction[6] and during the pilot experiments for this experiment. The reading pace was a compromise between being slow enough that the brain signals of two consecutive words to not overlap too much, and still being fast enough that a (more or less) fluent reading is possible.

Following the word stream, two extra sub-tasks were presented to validate that the participants had remembered their chosen word and that they had paid attention to both sentences. First, they were asked to type in the name of the relevant topic in order to ascertain they had not forgotten. Then, a recall task was presented to prevent the participants from selectively concentrating on one of the two sentences. One of the sentences was selected randomly and presented in full on the screen, with one of the nouns or verbs substituted by question marks. Participants were asked to type in the word missing in the sentence. They were then presented with feedback in points regarding their performance on these two tasks as a motivational instrument (similar to[7]).

Then, in the final part of the trial, the participants were asked to explicitly rate the relevance of all words from the relevant topic. All words were shown in one (if the sentence comprised fewer than 35 words) or two columns on the screen. A cursor was presented next to each word, indicating a two-alternative forced-choice decision. Pressing the left arrow key on the keyboard would rate the word as irrelevant and pressing the right would rate it as relevant. Participants were instructed prior to the experiment that they should not re-interpret the relevance of the words and instead make a decision based on their previous viewing of the sentence. To facilitate this, they received a maximum of 2 s to respond to each word, after which the cursor moved to the next word in the sentence. After the last word was rated, the trial was completed, with the next trial starting after an inter-trial interval of ca. 1 s, unless it was the last trial in the block.

After completing a block, they were requested to freely write about their chosen, relevant topic; this task was defined to keep the participant engaged. Finally, they filled out a questionnaire with two items for both topics, one regarding their interest ("how interesting do you find topic *x*") and one regarding their knowledge ("how much do you know about topic *x*") using a 9-point rating scale (1: not at all – 9: extremely so). Three self-timed breaks with a minimum of one minute evenly split the blocks into four parts. The experiment, excluding preparation and instruction, lasted approximately one hour.

Apparatus and Stimuli

Words were presented with an 18-point Lucida Console black typeface at the center of the 19" LCD screen. They were shown against a silver (RGB 82%, 82%, 82%) background in the middle of a 300×100 pixel pattern mask. The mask was a black rectangle with a grid-like pattern, with an opening to show the word. This was used to control the degree to which word length affected light reaching the eyes (i.e. to make sure longer words were not tantamount to more black pixels on the screen). Sentence separators were word-like character repetitions consisting of 4 to 9 numbers (3333333) or other non-alphabetic characters (&&&&&&), which were designed to mimic the same early visual activity as words without evoking psycholinguistic processing.

The screen was positioned approximately 60 cm from the participants and was running at a resolution of 1680 x 1050 and a refresh rate of 60 Hz. Stimulus presentation, timing, and EEG synchronization were controlled using E-Prime 2 Professional 2.0.10.353 on a PC running Windows XP SP3. EEG was recorded from 32 Ag/AgCl electrodes, positioned on standardized (using EasyCap elastic caps, EasyCap GmbH, Herrsching, Germany), equidistant electrode sites of the $10-20$ system via a QuickAmp (BrainProducts GmbH, Gilching, Germany) amplifier running at 200 Hz. Additionally, the electro-oculogram for vertical eye movements (and eye blinks) and horizontal eye movements was recorded using bipolar electrodes positioned respectively 2 cm superior/inferior to the right pupil and 1 cm lateral to the outer canthi of both eyes.

Pilot experiments

Prelimary versions of the final experimental procedure and design were piloted with four separate participants. In these experiments, we tested and evaluated, for example, the stimulus duration, the explicit feedback task, and the points system. The data of these pilot experiments were not used in the final analysis, except that some basic parameter estimations for the final feature engineering process were based on cross-validation experiments on these data (e.g., number of feature windows).

SI Data Analysis Details

The evaluation setup for prediction and retrieval followed the general block structure defined by the experimental design. We applied a participant-specific and leave-one-block-out learning and evaluation strategy. The individual prediction models are single-trial prediction models[8]. We report averaged prediction and retrieval performance, unless otherwise noted.

In detail, for a given participant, $B = \{1,...,8\}$ blocks with explicit term relevance judgments provided by the participant were available. In order to retrieve a *brain-feedback*-based list of relevant documents for a specific block b, two steps were executed. First, to obtain a term relevance prediction model for the given block b, a classification model f_b was trained using the data from the remaining $\{B \setminus b\}$ blocks. The prediction performance of f_b was then evaluated on the left-out block b. Second, to retrieve the set of documents for block b, the set of terms predicted to be relevant by the classifier f_b with a probability higher than 0.5 were used. The retrieval performance was evaluated against the expert judgements of document relevance for the relevant topic of block b.

As a baseline comparison, we evaluated the brain feedback-based performances against *random-feedback*-based performances. The random-feedback scenario corresponds to standard permutation tests and results in permutation-based p-values[9]. The following sections give concrete details on the methodology used.

EEG pre-processing

The EEG signals were cleaned and prepared following standard BCI guidelines[10]. During recording a hardware low-pass filter at 1000 Hz was applied. The continous EEG recordings were filtered with a 35 Hz FIR1 low-pass filter and a 0.5 Hz high-pass filter. The signal was then divided into epochs ranging from -250 ms to 1000 ms relative to the onset of each stimulus. Baseline correction was performed on each epoch using the pre-stimulus period. A simple heuristic was applied to reject invalid channels and epochs: First, invalid epochs were estimated based on the epochs' variances (< 0.5 μV) and the max-min criterium (40 μV). A channel was removed if the number of invalid epochs was higher than 10% of all available epochs. After removing all invalid channels, invalid epochs were estimated again and removed. This data cleaning approach was carried out in order to eliminate noise and potential confounds by common artifacts such as eye movements and blinks, as well as artefacts caused by loose electrodes or a cap that did not fit perfectly. Table 2 shows the statistics for the cleaning process for each participant.

Feature engineering

Event-related potentials are characterized by their temporal evolution and the corresponding spatial potential distributions. We followed standard feature engineering procedures to create spatio-temporal ERP features for classification[10]. For each epoch, the raw EEG data (after basic cleaning) were available as the spatio-temporal matrix $X^{m \times t'}$, with m channels and t' sampled time points. For each epoch, the time was divided into $t = 7$ equidistant windows between 250 ms and 950 ms after the stimulus onset. The number of windows was chosen based on data recorded during the pilot experiments. For each channel, the potential

values within one window were averaged, resulting in the spatio-temporal matrix $X^{m \times t}$. The final feature representation of one epoch was the concatenation of all columns into one vector $X^{m \cdot t}$. And, for a specific block b with n epochs, the full spatio-temporal feature matrix used for classification was $\underline{X}^{n \times m \cdot t}$. Note that the number of channels m and the number of epochs n were participant-specific, as they were dependent on the EEG cleaning and preparing procedure. On average, the spatio-temporal feature matrix consisted of 25 channels $\times$ 7 windows $=$ 175. Table 2 shows the concrete numbers for each participant.

SI Term Relevance Prediction

We developed term relevance prediction models within the framework of the linear EEG model[11] and single-trial ERP classification[10]. In detail, we utilized Linear Discriminant Analysis (LDA, see[12]) and learned linear binary classifiers, which we used to predict class memberhsip probabilities. The assumptions of the method are that the observations X have been drawn from two multivariate Normal distributions $N(\mu_k, \Sigma)$, one for the class of "relevant" observations, and the other for the class of "irrelevant" observations. For the estimation of the models we used shrinkage LDA, a covariance-regularized LDA with a shrinkage parameter selected by the analytical solution developed by Schäfer and Strimmer[13]. The choice of this simple method was based on the many existing successful applications using this method in the BCI community[10]. In addition, one major reason is robustness against class imbalance[14], an obvious situation in the proposed paradigm (see also Table 2 for the relevance class distribution per participant).

Leave-one-block-out evaluation

For each participant, we trained a set of eight classifiers. The classifer f_b for block b was trained with the epochs from the other blocks, i.e., with the spatio-temporal feature matrix $\underline{X}_{\{B \setminus b\}}^{n_l \times m \cdot t}$. The classifier f_b was evaluated on the epochs from block b, i.e., on the matrix $\underline{X}_b^{n_t \times m \cdot t}$. The performance measures of interest were the *Area under the ROC curve* (AUC), *precision*, and *tf-idf*-weighted precision. The AUC is defined as the area under the ROC curve, which links the true positive rate to the false positive rate. A perfect model has an AUC of 1, and a random model has an AUC of 0.5. AUC is a global quality measure of the classification model. This measure was chosen because it allowed us to correctly evaluate the models in the existing class imablance scenario and because it is a comprehensive measure for comparison to the random feedback models. Precision is defined as

$$\mathrm{tp}/(\mathrm{tp}+\mathrm{fp}), \tag{1}$$

where tp is the number of true positives (i.e., relevant words predicted to be relevant) and fp is the number of false positives (i.e., irrelevant words predicted to be relevant). This measure was chosen because we want to have a high precision (i.e., many correct relevant words) for the document retrieval step. Weighted precision is defined as

$$(w_{\mathrm{tp}} * \mathrm{tp})/(w_{\mathrm{tp}} * \mathrm{tp} + w_{\mathrm{fp}} * \mathrm{fp}), \tag{2}$$

where w_{tp} is the sum of the term-frequency–inverse document frequency (*tf-idf*) values of the true positive words, and w_{fp} is the sum of tf-idf values of the false positive predicted words. In our case, the *tf-idf* values either come from the relevant document or the irrelevant document. For a positive predicted word that is not available in a document, the *tf-idf* is set to 0. This reflects that this word has no influence on the document retrieval.

Random feedback evaluation

For a given block b, a classifier was trained and evaluated on data with permuted relevance judgments. If executed for a large number of permutations, this random-feedback strategy is a permutation test, resulting in a permutation-based p-value[15]. The null hypothesis of the test assumes that the brain data and the relevance judgments are independent. A small p-value indicates that the classifier is able to find a significant structure discriminating "relevant" and "irrelevant" brain signal patterns. For each block, $k = 1000$ permutations were performed, meaning that the smallest possible p-value is 0.001[9].

SI Intent Modeling-based Recommendation

We developed an intent estimation model to predict how relevant each term the user read is to the topic of interest. This model was then used to retrieve new documents from the database. The motivation for the intent model is that the predictions of the term-relevance model can indicate the relevance to a topical intent, but the individual words for which the predictions are drawn may not represent the whole topic. For example, the words "matter" and "neutrons" are related to the topic "Atom," but would not alone be sufficient search terms to retrieve information about the topic "Atom." Therefore, these words are used as positive feedback for the intent model to predict that, for example, the words "atom," "atomic," and "nucleus" are also relevant for the user given the positively predicted words "matter" and "neutrons." We call the resulting model the intent model of the user[16].

Document representation

The documents and words are modeled as a term-document matrix K with i terms and j documents. The term vector k_i indicates the weight of a stemmed word for each of the documents. The words are stemmed, i.e., reduced to its stem, base, or root, using the English Porter Stemmer[17]. For example, the word "cats" is reduced to its root "cat", or the word "playing" is reduced to its root "play". The stemmed words are referred to as terms. Before stemming, English stop words were removed. Stop words are typically the most common words of a languages, e.g., "the", "and", and "a". We used the stop word definition provided in the Apache Lucence 4.10 stop word list[1]. We used tf-idf weighting to account for the frequency and specificity of each term[18]. The *tf-idf*, which stands for term frequency-inverse document frequency, is a weight often used in information retrieval to statistically measure how important a word is to a document in a collection or corpus. We use a logarithmically scaled tf-idf formally defined as:

$$tf - idf = tf \cdot idf \tag{3}$$

such that

$$tf = 1 + \log(f_{t,d}) \tag{4}$$

and

$$idf = \log(1 + \frac{N}{n_t}) \tag{5}$$

, where $f_{t,d}$ is the frequency of the term t occurring in the document d, N is the total number of documents in the corpus, and n_t is the total number of documents where the term t appears.

Intent model

The intent model estimates a weight for each term based on the input from the term-relevance prediction classifier. The feedback from the term-relevance predictions is denoted as $r_i \in [0,1]$ for a subset of terms indexed by i. We assume that the term-relevance prediction r_i of a term k_i is a random variable with expected value $E[r_i] = k_i \cdot w$, such that the expected weight is a linear function of the terms. The unknown weight vector w is essentially the representation of the user's intent and determines the relevance of terms.

To estimate w we utilize the LinRel algorithm[19]. It learns a linear regression model of the form $r = wK$. LinRel allows control for the uncertainty related to the term weight estimates. The choice of this method was based on its robustness against suboptimal input, which is the case for potentially noisy predictions of the term-relevance prediction model.

LinRel computes a regularized regression weight vector for each term k_i in K:

$$a_i = k_i(K^\top K + \lambda I)^{-1} K^\top, \tag{6}$$

where I is the identity matrix, and λ is a regularization parameter set to 0.5, and all terms except k_i on the right-hand side are shared for all keywords. Then for each keyword, the final relevance score w_i at the current iteration is computed by taking into account the feedback obtained so far:

$$w_i = a_i \cdot s_t + \frac{c}{2}\|a_i\|, \tag{7}$$

where s_t is the vector of term-relevance predictions obtained, a_i is the weight vector of a single keyword i in the data K, $\|a_i\|$ is the L_2 norm of the weight vector, and the constant c is used to adjust the influence of the history (we used $c = 2$ to give equal weight for exploration and exploitation). It can be shown that this procedure is equivalent to estimating the upper confidence bound in a linear regression problem[19].

Retrieval model

Intent model estimates a weight w for each term which, in turn, is used to retrieve new documents from the database, to be recommended for the user. We use a unigram language modeling approach of information retrieval[20]. In detail, the vector w is treated as a sample of a desired document, and documents d_j are ranked by the probability that w would be generated by the respective language model M_{d_j} for the document d_j.

Using maximum likelihood estimation, we get

$$P(w|M_{d_j}) = \prod_{i=1}^{|\mathbf{w}|} \hat{P}_{mle}(k_i|M_{d_j})^{w_i}, \tag{8}$$

[1] https://lucene.apache.org/

and to avoid zero probabilities and improve the estimation we then compute a smoothed estimate by Bayesian Dirichlet smoothing so that

$$\hat{P}_{mle}(k_i|M_{d_j}) = \frac{c(k_i|d_j) + \mu p(k_i|C)}{\sum_k c(k|d_j) + \mu}, \tag{9}$$

where $c(k|d_j)$ is the count of term k in document d_j, $p(k_i|C)$ is the occurrence probability (proportion) of term k_i in the whole document collection, and the parameter μ is set to 2000 as suggested in the literature[21].

Recommendation evaluation

The evaluation setup for the recommendation was designed analogously to term-relevance prediction. Each classifier output f_b for a block b was given as input for the intent model. The resulting intent model was used to predicted relevant words, and a ranked set of the top-30 documents were retrieved from the whole English Wikipedia corpus.

Random feedback recommendation

For a given block b, the recommendation was evaluated with term-relevance input resulting from permuted relevance judgments. Similarly to the relevance prediction, this random strategy is also a permutation test. A small p-value indicates that the recommendation system is able to gain more relevant documents based on the brain input than with the random input. Following the evaluation setup of term-relevance prediction, $k = 1000$ permutations were performed for each block.

Performance measures

The recommendation performance was evaluated using *Cumulative information gain* (CG)[22]. The cumulative information gain is defined simply as the sum of the relevance scores assigned by the experts for the documents that were ranked in the top-30 documents by the retrieval system in response to the input. Formally,

$$CG = \sum_{30}^{i=1} rel_i, \tag{10}$$

where rel_i is the relevance score of the ith document in the ranked list. This measure was chosen because it allows graded relevance assessments: some documents may be highly relevant and some documents may be marginally relevant. The cumulative gain may be different for different topics: some topics may have many highly relevant documents, and some may have only a few.

References

1. Wikimedia. Wikimedia downloads (2014). Https://dumps.wikimedia.org/.
2. Cohen, J. Weighted kappa: Nominal scale agreement provision for scaled disagreement or partial credit. *Psychological Bulletin* **70**, 213–220 (1968).
3. Oldfield, O. R. The assessment and analysis of handedness: The Edinburgh inventory. *Neuropsychologia* **9**, 97–113 (1971).
4. Cohen, M. S. Handedness questionnaire (2014). Http://www.brainmapping.org/shared/Edinburgh.php.
5. Cambridge English Language Assessment. Test your English – Adult Learners (2014). Http://www.cambridgeenglish.org/test-your-english/adult-learners/.
6. Eugster, M. J. A. *et al.* Predicting term-relevance from brain signals. In *Proceedings of the 37th International ACM SIGIR Conference on Research and Development in Information Retrieval*, SIGIR '14, 425–434 (ACM, New York, NY, USA, 2014).
7. Spapé, M. M., Band, G. P. & Hommel, B. Compatibility-sequence effects in the Simon task reflect episodic retrieval but not conflict adaptation: Evidence from LRP and N2. *Biological psychology* **88**, 116–123 (2011).
8. Blankertz, B., Curio, G. & Müller, K.-R. Classifying single trial eeg: Towards brain computer interfacing. In Dietterich, T., Becker, S. & Ghahramani, Z. (eds.) *Advances in Neural Information Processing Systems 14*, 157–164 (MIT Press, 2002).
9. Good, P. I. *Permutation Tests: A Practical Guide to Resampling Methods for Testing Hypotheses* (Springer, 2000), 2 edn.
10. Blankertz, B., Lemm, S., Treder, M., Haufe, S. & Müller, K.-R. Single-trial analysis and classification of ERP components – A tutorial. *NeuroImage* **56**, 814–825 (2011).
11. Parra, L. C., Spence, C. D., Gerson, A. D. & Sajda, P. Recipes for the linear analysis of EEG. *NeuroImage* **28**, 326–341 (2005).

12. Hastie, T., Tibshirani, R. & Friedman, J. *The Elements of statistical learning* (2009), 2 edn.

13. Schäfer, J. & Strimmer, K. A shrinkage approach to large-scale covariance matrix estimation and implications for functional genomics. *Statistical Applications in Genetics and Molecular Biology* **4** (2005).

14. Xue, J.-H. & Titterington, D. M. Do unbalanced data have a negative effect on LDA? *Pattern Recognition* **41**, 1558–1571 (2008).

15. Ojala, M. & Garriga, G. C. Permutation tests for studying classifier performance. *Journal of Machine Learning Research* **11**, 1833–1863 (2010).

16. Ruotsalo, T. *et al.* Directing exploratory search with interactive intent modeling. In *Proceedings of the 22nd ACM International Conference on Information and Knowledge Management*, CIKM '13, 1759–1764 (ACM, New York, NY, USA, 2013).

17. Porter, M. F. An algorithm for suffix stripping. In Sparck Jones, K. & Willett, P. (eds.) *Readings in Information Retrieval*, 313–316 (Morgan Kaufmann Publishers Inc., San Francisco, CA, USA, 1997).

18. Jones, K. S. A statistical interpretation of term specificity and its application in retrieval. *Journal of Documentation* **28**, 11–21 (1972).

19. Auer, P. Using confidence bounds for exploitation-exploration trade-offs. *J. Mach. Learn. Res.* **3**, 397–422 (2003).

20. Ponte, J. M. & Croft, W. B. A language modeling approach to information retrieval. In *Proceedings of the 21st Annual International ACM SIGIR Conference on Research and Development in Information Retrieval*, SIGIR '98, 275–281 (ACM, New York, NY, USA, 1998).

21. Zhai, C. & Lafferty, J. A study of smoothing methods for language models applied to ad hoc information retrieval. In *Proceedings of the 24th Annual International ACM SIGIR Conference on Research and Development in Information Retrieval*, SIGIR '01, 334–342 (ACM, New York, NY, USA, 2001).

22. Järvelin, K. & Kekäläinen, J. Cumulated gain-based evaluation of IR techniques. *ACM Trans. Inf. Syst.* **20**, 422–446 (2002).

Step-by-step explanation

Beginning of a block:

Screen	Comment
Choose a topic<br>• cat<br>• football	*A block of Part 1 starts; Select one of the two topics.*
Please keep in mind: cat.<br>Count the words that are RELEVANT.<br>After that, remember: cat	*Instructions: Read what to do in this block:*<br>• *Count relevant words (relevant **for the topic you selected**)*<br>• *Just read*

Trial 1 begins

Screen	Comment
Sentence: the	*First word of the first sentence*
cat	*Second word…*
…(more words)..	
######	*A separator is shown to indicate the other topic begins*
association	*First word of the second sentence*
football	*Second word…*
…(more words)..	
######	*A separator is shown to indicate the other topic begins*
Which word were you to remember?	*Question about the topic: type the relevant topic, here "cat"* [1].
How many words were relevant?	*Question about the task: type the number of relevant words you counted*[1].
Which word is missing:<br>*The domestic cat is a small, usually furry, ??????, and carnivorous mammal.*	*Question about one of the two sentences: Fill in the missing word, here "domesticated"* [1].
Points:<br>1) Remembering: -1 (wrong) or +1 (right)<br>2) The number of relevant:<br>0 (wrong), +1 (ok), +2 (very good)	*Info screen about the score you got for this trial*
You will see the words again. Please rate the relevance by pressing left and right.	*Info screen about the relevance rating.*
the<br>cat<br>is<br>…	*Rate each word by using the left or right arrow. Left is irrelevant, right is relevant.*<br>*You cannot change the rating!*

Trials 2 to 6 in the same way.

After 6 sentences, the block ends and a questionnaire starts:

Screen	Comment
How much do you **know** about the topic cat? Please press a key to indicate the answer (1 Nothing – 9 Everything).	*Indicate knowledge about relevant topic.*
How **interesting** do you find cat?	*Indicate interest.*
How much do you **know** about football?	*Indicate knowledge (other topic)*
How **interesting** do you find football?	*Indicate interest*

Only if the task was **"Just read", you will be asked to write something about the topic you chose:**

Screen	Comment
Write something about "cats" you learned in this block.	*We will do a lottery amongst the best answers we found here.*

1 Answer as fast as possible!

Figure 1. Step-by-step explanation of the experiment. The illustrations shows the composition of one reading task (block). A participant conducted eight such reading tasks with each one with different documents. This explanation was part of the information sheet all participants received during their initial briefing.

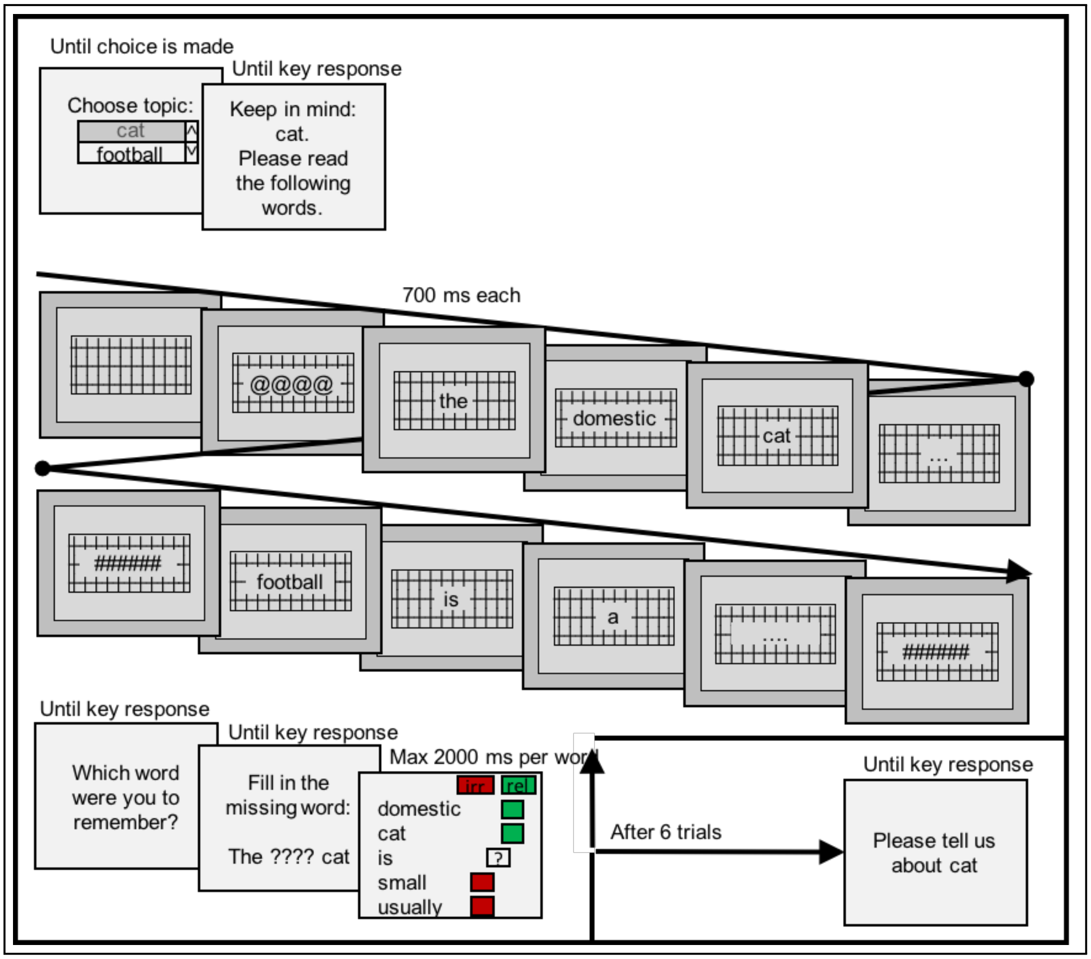

Figure 2. Illustration of the technical implementation as a cognitive neuroscience experiment. The figure shows the screen by screen execution of the block described in Figure 1.

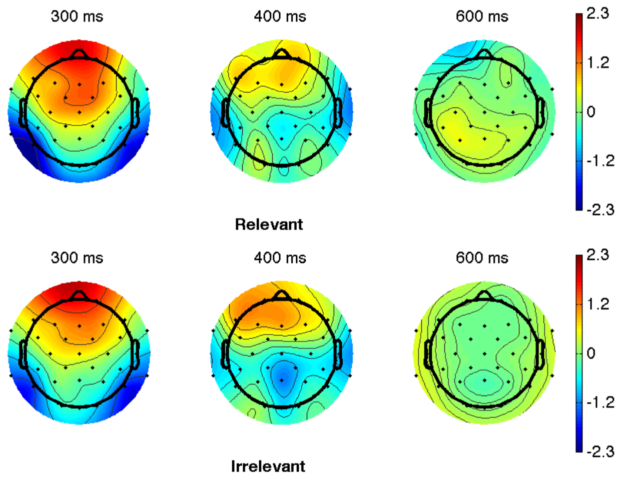

Figure 3. Grand average-based topographic skalp plots of relevant words (top) and irrelevant words (bottome) for different time windows.

Document	#Relevant	#Irrelevant	#Retrieved Documents	#Relevant Documents	#Irrelevant Documents	Top-30 Score	Maximum Score
Association football	5	4	470	34	436	52	56
Atom	7	1	461	56	405	68	94
Automobile	5	4	477	40	437	36	46
Bank	4	4	537	47	490	47	64
Bicycle	2	5	380	35	345	53	58
Bill Clinton	2	5	306	55	251	48	73
Brain	5	0	257	46	211	47	63
Cat	7	4	545	55	490	66	91
Communism	3	4	428	43	385	47	60
Euro	2	3	288	41	247	63	74
India	6	0	468	120	348	90	244
Learning	4	5	497	81	416	70	125
Machine Learning	6	2	491	51	440	89	118
Michael Jackson	3	5	517	54	463	90	147
Money	4	5	478	123	355	90	249
Ocean	5	5	426	79	347	90	167
Painting	3	8	617	51	566	90	136
Plato	3	3	337	94	243	90	185
Politics	5	6	588	172	416	90	337
Rome	3	5	474	62	412	90	150
Savanna	1	6	471	41	430	47	58
Schizophrenia	6	3	484	51	433	69	90
School	2	6	414	30	384	51	51
Society	5	6	688	79	609	53	102
Star	4	5	524	98	426	64	132
Telephone	2	3	354	44	310	60	74
Time	6	2	525	56	469	59	85
Volcano	4	3	442	76	366	60	106
Wife	2	5	450	66	384	49	85
Wine	4	3	577	89	488	90	183
Total	120	120	13971	1969	12002	2008	3503

Table 1. Description of the 30 documents used in the experiment. The first column shows the name of the document. The second and third columns show how often the document was presented to the users and how often it then was chosen as relevant or irrelevant. The fourth column shows the number of retrieved documents for a given document pooled over all experiments. The fifth and sixth columns show how many of the retrieved documents were judged by the experts to be relevant or irrelevant given the topic. The seventh column shows the sum of the relevance scores of the top-30 documents. The seventh column shows the sum of all relevance scores.

Participant	#Recorded Channels	# Accepted Channels	#Blocks	#Recorded Epochs	#Accepted Epochs	#Relevant Epochs	#Irrelevant Epochs
TRPB101	32	26	8	1941	1376	153	1223
TRPB102	32	26	8	1961	1659	193	1466
TRPB103	32	11	8	1936	1521	242	1279
TRPB105	32	30	8	1986	1521	198	1323
TRPB106	32	29	8	1959	1486	215	1271
TRPB107	32	20	8	1960	1566	245	1321
TRPB109	32	30	8	1869	1622	315	1307
TRPB110	32	20	8	1958	1021	103	918
TRPB111	32	31	8	1818	1045	170	875
TRPB112	32	30	8	2026	1588	268	1320
TRPB113	32	26	8	1939	1422	195	1227
TRPB114	32	26	8	1944	1226	204	1022
TRPB115	32	30	8	1896	1441	211	1230
TRPB116	32	28	8	1981	1662	242	1420
TRPB117	32	16	8	1906	1364	326	1038

Table 2. Description of the EEG recordings. The first column shows the identifier of the participant. The second and third columns show the number of recorded and the number of accepted channels after cleaning per participant. The fourth column shows the number or blocks recorded for each participant. The fifth and sixth columns show the number of recorded and the number of accepted epochs after cleaning per participant. The seventh and eighth columns show the number of relevant and irrelvant epochs.

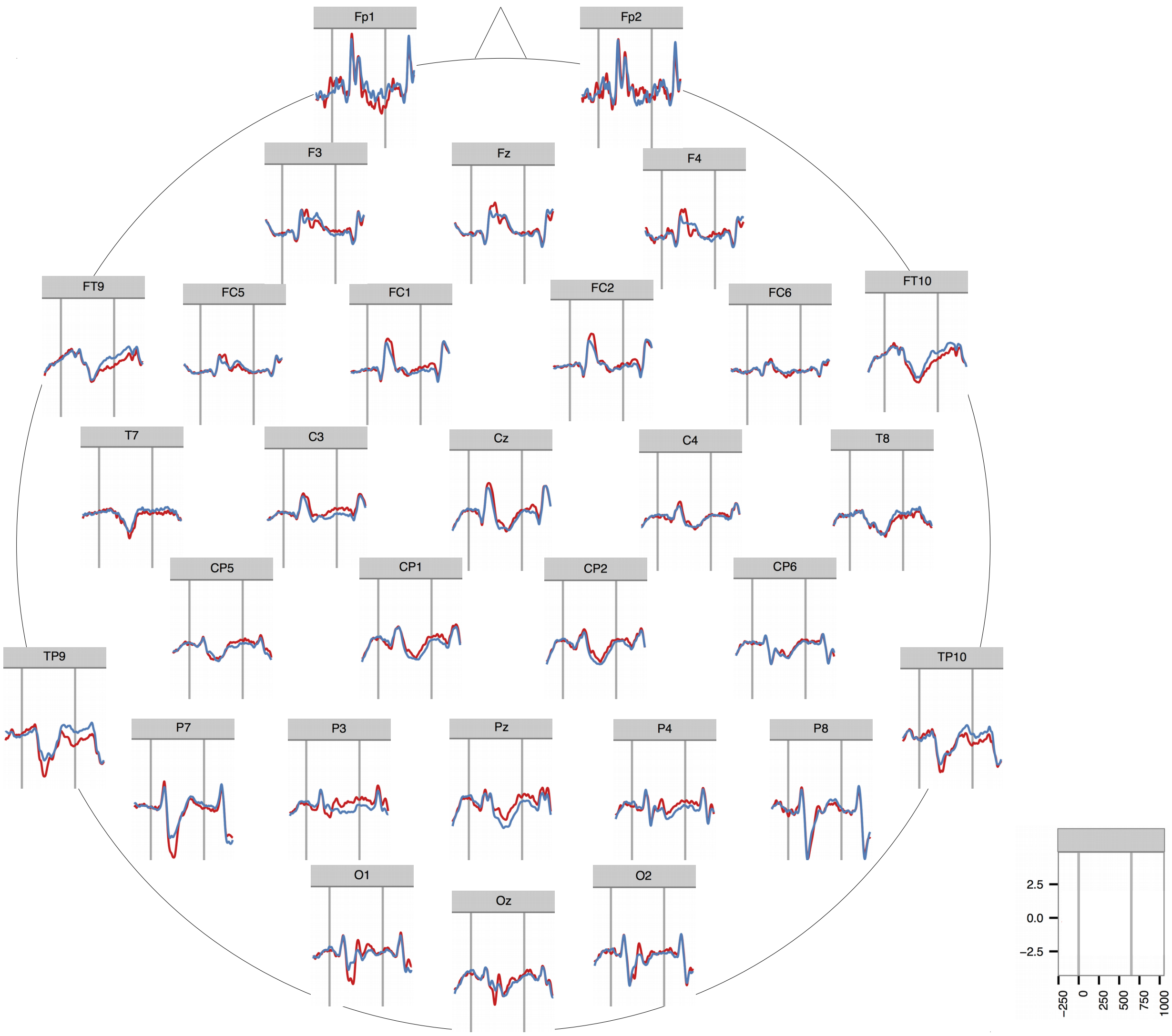

Figure 4. Grand average event-related potential at all channels of relevant (red curves) and irrelevant (blue curves) terms. The gray vertical lines show the word onset events.

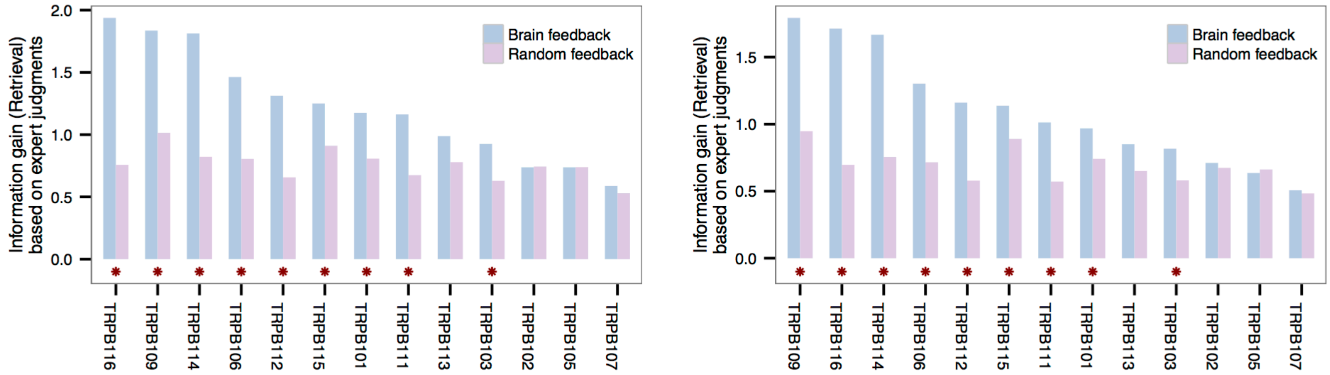

Figure 5. Average cumulative information gain (on a scale 0-3) based on the Top-10 (left) and the Top 20 (right) retrieved documents for the participant. Asterisks indicate a significantly better pooled information gain based on brain feedback than randomized feedback retrieval on 1000 iterations.

Participant	p-value
TRPB101	0.0030
TRPB102	0.0010
TRPB103	0.0010
TRPB105	0.0090
TRPB106	0.0010
TRPB107	0.0010
TRPB109	0.0010
TRPB110	0.8541
TRPB111	0.0010
TRPB112	0.0010
TRPB113	0.0040
TRPB114	0.0010
TRPB115	0.0050
TRPB116	0.0010
TRPB117	0.1439

Table 3. Test statistics for the tests results shown in Figure 5. For each participant a permutation test with 1000 iterations was executed. In each iteration, the relevance judgments were permutated. The p-value is then based on the number of times the randomized classification is better than the brain feedback-based classification with respect to the AUC values.

Participant	W	p-value
TRPB101	112811.5000	0.0009
TRPB102	111581.0000	0.9573
TRPB103	99082.5000	0.0001
TRPB105	104242.0000	0.4398
TRPB106	119899.0000	0.0000
TRPB107	94403.5000	0.9789
TRPB109	138593.5000	0.0000
TRPB111	136092.5000	0.0000
TRPB112	135740.0000	0.0000
TRPB113	127788.5000	0.0052
TRPB114	166286.0000	0.0000
TRPB115	110133.5000	0.0031
TRPB116	165508.0000	0.0000

Table 4. Test statistics for the tests results shown in Figure 7. For each participant a two-sided Wilcoxon test was executed between the brain feedback-based retrieved document scores and the random feedback-retrieved document scores.
